# Risk factors for chronic kidney disease in Japan: a community-based study

**DOI:** 10.1186/1471-2369-10-34

**Published:** 2009-10-27

**Authors:** Norimichi Takamatsu, Hideharu Abe, Tatsuya Tominaga, Kunihiko Nakahara, Yumi Ito, Yoko Okumoto, Jiyoong Kim, Masafumi Kitakaze, Toshio Doi

**Affiliations:** 1 Department of Clinical Biology and Medicine, Graduate School of Medicine, Institute of Health-Bio-Science, University of Tokushima, Tokushima, Japan; 2 Hubit Genomix Inc., Tokyo, Japan; 3 Arita-cho, Saga, Japan; 4 National Cardiovascular Center, Osaka, Japan

## Abstract

**Background:**

Chronic kidney disease (CKD) is increasingly being recognized as a predictor for both end-stage renal disease and cardiovascular disease. The present study, conducted on individuals from a community in Arita, Japan, was designed to evaluate biomarkers that can be used to determine the associated factors for CKD.

**Methods:**

This study involved 1554 individuals. Kidney function was evaluated in terms of the creatinine-based estimated glomerular filtration rate (eGFR), which was determined using the Modification of Diet in Renal Disease equation. Low eGFR was defined as eGFR < 60 mL/min per 1.73 m^2^. The concentration of both urinary albumin and urinary type IV collagen were measured.

**Results:**

In the younger participants (age, <65 years), the odds ratio (95% confidence interval [CI]) of low eGFR was 1.17 (1.02 to 1.34) for each 1 year older age, 6.28 (1.41 to 28.03) for urinary albumin creatinine ratio (ACR) over 17.9 mg/g and 9.43 (2.55 to 34.91) for hyperlipidemia. On the other hand, among the elderly participants (age, ≥ 65 years), the odds ratio (95% CI) of low eGFR was 2.97 (1.33 to 6.62) for gender, 1.62 (1.06 to 2.50) for hypertension and 1.97 (1.19 to 3.28) for hyperlipidemia. Urinary type IV collagen creatinine ratio was not identified as an associated factor for low eGFR.

**Conclusion:**

In this present cross-sectional community-based study, ACR is associated with CKD, which was defined as an eGFR of less than 60 mL/min per 1.73 m^2^, in the younger participants but not in the older participants.

## Background

The frequency of end-stage renal disease (ESRD) has been increasing rapidly not only in Japan but also worldwide [1]. Several reports have shown that over the past 2 decades, the number of ESRD patients who require dialysis or transplantation has more than doubled in Europe and the United States during the past 2 decades [2-4]. Similarly, in Japan, the number of ESRD patients has been increasing continuously over the past 3 decades. In 2005, 36,063 ESRD patients (average age, 66 years) enrolled in a new dialysis program [5]. The incidences of renal diseases, especially chronic kidney disease (CKD) and ESRD, are increasing in an aging society, such as that in Japan [6]. CKD is an important risk factor not only for ESRD but also for cardiovascular disease [7-9]; therefore, the care for the individuals affected by CKD has become a matter of concern. Further, there is an increased societal demand for the identification of risk factors for CKD and for the development of strategies for the prevention of CKD.

Study findings on the prevalence of CKD vary considerably [10,11], and there are sparse data in this regard for Japanese populations. Diabetic nephropathy is the leading cause of CKD and ESRD, and urinary albumin was originally used as a biomarker for the detection of CKD in diabetic patients. In addition, the excretion of urinary type IV collagen (U-Col4) increases in diabetic patients with albuminuria [12]. The severity of renal insufficiency increases in elderly people; therefore, it is important to detect CKD and initiate treatment for this disease as early as possible. It is also necessary to assess renal dysfunction in elderly patients and to evaluate its associated risk factors using parameters such as urinary albumin and U-Col4 in normal and CKD-affected Japanese populations.

The present study was conducted in the town of Arita, which is a rural area in Japan. We investigated the association between renal function and the urinary excretion of albumin and U-Col4 and other confounding factors. In addition, we assessed the associated factors for low estimated glomerular filtration rate (eGFR) in different age groups (aged <65 years and ≥ 65 years). This community-based study investigates whether the urinary albumin creatinine ratio (ACR) and U-Col4 creatinine ratio (U-Col4CR) are associated factors for CKD in Japan.

## Methods

### Study sample and design

In 2002, an annual health examination was conducted in the town of Arita in Japan to investigate the cause and to find out early phase of lifestyle oriented disease. Arita-cho is located in the west of Saga Prefecture on the island of Kyushu in southern Japan. One of the main occupations in this town is agriculture and pottery (Arita-yaki), which is a popular industry in Japan.

The population of this town was approximately 9300, the population of males and females was 48.0% and 52.0%, respectively. We sent an official letter regarding an annual health examination and this program for the resident aged = 18 at first. We then sent a second letter of invitation for the resident who had not rejected this program. Finally, a total of 1564 people aged = 18 took part in the program and agreed to join the study. Ten participants were excluded from the present analysis due to incomplete data. A total of 1554 people aged 20-92 years entered into the final statistical analysis. There were 573 males (63.8 ± 14.2 years) and 981 females participants (62.6 ± 14.4 years).

This study is a community-based and baseline survey that consisted of a self-administered questionnaire on lifestyle, medical histories, anthropometrical measurements, and collections of blood and urine specimens from the participants.

### Questionnaires

Participants used a self-report questionnaire to document medical history and lifestyle. They answered whether they had ever been diagnosed with any of the following: heart disease, cerebral infarction, hypertension, renal disease, diabetes mellitus, anemia, hepatic disease, hyperuricemia, or hyperlipidemia. They answered alcohol intake (drinker or non-drinker), smoking habit (smoker or non-smoker), insufficient exercise, exercise habit (doing physical activity with sweat or not), regular meal consumption and eating speed (self-judgment as a normal speed eater or fast eater). Changing of weight from 20 years old was defined in two groups (not changed, changed; getting lighter or heavier). Fatty meal was defined in two groups (excess, not excess). Salty meal was classified into 3 groups (light, moderate, and heavy).

### Laboratory methods and estimation of the glomerular filtration rate

Blood was sampled from all the participants for the assessment of laboratory parameters. Fresh serum samples were analyzed. Serum creatinine (Cr) was measured using the uncompensated rate Jaffe reaction and an OLYMPUSAU5431 auto analyzer (Tokyo, Japan), and the normal values were found to range from 0.8 to 1.3 mg/dL and from 0.6 to 1.0 mg/dL for male and female, respectively.

We then estimated the glomerular filtration rate (GFR) by using the new 4-variable Modification of Diet in Renal Disease (MDRD) equation as follows [13-15]:

Participants with decreased kidney function were defined primarily by the presence of eGFR <60 mL/min per 1.73 m^2 ^using the simplified MDRD study equation. Individuals with decreased kidney function were classified further according to the CKD staging suggested by the guidelines of the standard Kidney Disease Outcomes Quality Initiative (K/DOQI) [15]. Individuals with low eGFR (eGFR 15 to 59 mL/min per 1.73 m^2^) were considered to have CKD.

Body mass index (BMI) was calculated by dividing weight (in kg) by height squared (in m^2^). Abnormal BMI level was set as ≥ 25 kg/m^2 ^both in males and females. The glycated hemoglobin (HbA_1c_) levels were measured by high performance liquid chromatography, and the normal values were found to range from 4.3% to 5.8%. The serum levels of total cholesterol, triglycerides and high-density lipoprotein in (HDL) cholesterol were determined enzymatically. Abnormal values were set as a total cholesterol level of >5.69 mmol/L (>220 mg/dL), a HDL cholesterol level of <1.03 mmol/L (<40 mg/dL), and/or serum triglyceride level of >1.69 mmol/L (>150 mg/dL). The serum aspartate amino transferase (AST) level of >40 U/L, alanine amino transferase (ALT) level of >40 U/L, and γ-glutamyl transpeptidase (γ-GT) level of >60 U/L was set as abnormal in males and females. Systolic and diastolic blood pressures were measured by using an automatic oscillometric method (OMRON; HEM-780) in the sitting position after at least 5 minutes rest. Measurement was performed twice, with the mean value used for statistical analysis. Abnormal values were defined as systolic blood pressure level of ≥ 140 mm Hg, diastolic blood pressure level of ≥ 90 mm Hg.

Urine samples were collected from the participants at their respective homes and transported to the laboratory. Proteinuria was defined as positive dipstick result by using Uro-Paper II EIKEN (Eiken-Chemical, Tokyo, Japan). First, the urinary Cr concentration was measured by performing an enzymatic method (Shino-Test, Tokyo, Japan).

Second, the urinary albumin concentration was measured by performing a turbidimetric immunoassay (Wako, Osaka, Japan), and ACR was calculated as the urinary albumin concentration divided by the urinary Cr concentration in order to exclude the influence of the urinary volume. Microalbuminuria and macroalbuminuria were defined as ACR 30-300 mg/g and >300 mg/g, respectively.

Third, the U-Col4 level was measured by performing a one-step sandwich enzyme immunoassay using 2 monoclonal antibodies against the 7S domain and the other against the triple helix (TH) domain (Panauria uIV C; Daiichi Fine Chemical Co Ltd., Takaoka, Japan). U-Col4CR was calculated as the U-Col4 concentration divided by the urinary Cr concentration in order to compensate for the urinary volume.

We calculated the normal cut-off range of ACR and U-Col4 for 442 healthy adults (age, 20-70 years) who yielded normal results on both physical examination and laboratory analyses. For statistical analysis, data are reported as means ± S.D. and the 95th percentile was used. The normal cut-off range for the ACR was found to be less than 17.9 mg/g and for U-Col4CR was less than 8.2 μg/g and 9.4 μg/g for males and females, respectively.

### Statistical analysis

In the analysis of the baseline characteristics, continuous variables of normal distribution were evaluated for statistical significance across the GFR grade using ANOVA. A χ^2 ^test was used for statistical comparisons of the GFR grade and the abnormal rate noted for each variable assessed in the participants. Both univariate and multivariate logistic regression analyses were performed to assess the correlation among the risk factors identified for each classified age group and the gender, BMI, HbA_1c_, ACR, U-Col4CR, hypertension, and hyperlipidemia, alcohol intake and smoking habit. The odds ratio (95% confidence intervals [CIs]) was calculated using a multivariate logistic regression analysis. A *p *value of less than 0.05 was considered statistically significant. Statistical analyses were performed with SPSS version 14.0 (SPSS, Chicago, IL).

### Ethical approval

This study was approved by the Ethics Committee of the Arita-cho, HuBit genomics, Inc. and was performed in compliance with the Helsinki Declaration. Written informed consent was obtained from all participants.

## Results

### eGFR Distribution

**Figure 1:**
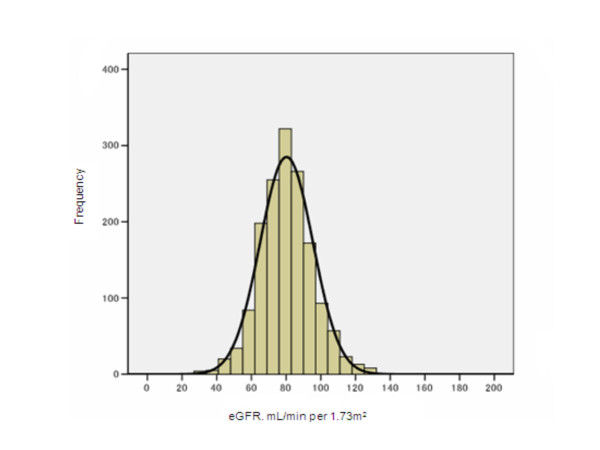
**Distribution of eGFR in study participants**. The histogram of eGFR from 1554 participants indicates a normal distribution of the group and the average of eGFR is 80.2 with a standard deviation of 15.2 mL/min/1.73 m^2^.

The eGFR was evaluated in 1554 participants; the histogram in Figure 1 shows standard deviation. The average eGFR and standard deviation values for all participants were 80.2 mL/min per 1.73 m^2 ^and 15.22, respectively (those for males were 80.6 mL/min per 1.73 m^2 ^and 14.80, respectively and females, 80.0 mL/min per 1.73 m^2 ^and 15.46, respectively).

### Characteristics of the study participants among the groups formed on the basis of the eGFR

The baseline characteristics of the participants are shown in Table 1. The mean values observed for BMI, HbA_1c_, hemoglobin, serum cholesterol, triglyceride, and HDL cholesterol levels, systolic and diastolic blood pressure, ACR, and U-Col4CR were significantly different among the 3 groups.

**Table 1:** Characteristics of the study participants among the groups divided by eGFR*

	eGFR, mL/min/1.73 m^2^			
	
	15--59	60--89	≥ 90	p value
Participants, n (%)	130 (8.4)	1058 (68.1)	366 (23.5)	
Male sex, %	29.2	36.9	39.6	0.108
Mean age (SD), y	73.0 (8.0)	65.7 (12.1)	51.7 (15.6)	< 0.001
Mean body mass index (SD), kg/m^2^	22.7 (3.3)	22.5 (3.0)	21.7 (3.3)	< 0.001
Mean HbA_1c _(SD), %	5.45 (0.6)	5.25 (0.7)	5.14 (0.8)	< 0.001
Mean hemoglobin level (SD),				< 0.001
mmol/L	7.89 (0.90)	8.37 (0.87)	8.33 (1.04)	
mg/dL	12.71 (1.45)	13.48 (1.40)	13.42 (1.68)	
Mean serum cholesterol level (SD),				< 0.001
mmol/L	5.24 (0.859)	5.14 (0.819)	4.79 (0.768)	
mg/dL	202.6 (33.2)	198.96 (31.7)	185.3 (29.7)	
Mean serum triglyceride level (SD),				< 0.001
mmol/L	1.28 (0.640)	1.19 (0.652)	1.00 (0.598)	
mg/dL	113.7 (56.7)	105.3 (57.7)	88.2 (53.0)	
Mean serum HDL cholesterol level (SD),				< 0.001
mmol/L	1.35 (0.331)	1.38 (0.313)	1.45 (0.333)	
mg/dL	52.1 (12.8)	53.5 (12.1)	56.1 (12.9)	
Mean AST (SD), U/L	25.6 (8.9)	26.3 (40.7)	23.4 (14.1)	0.399
Mean ALT (SD), U/L	19.2 (9.9)	24.4 (84.5)	20.9 (13.0)	0.577
Mean γ-GT (SD), U/L	32.8 (32.6)	39.9 (54.7)	41.5 (64.0)	0.303
Mean serum creatinine level (SD),				< 0.001
μmol/L	91.5 (15.26)	67.9 (7.63)	57.2 (7.63)	
mg/dL	1.20 (0.2)	0.89 (0.1)	0.75 (0.1)	
Mean systolic blood pressure (SD), mmHg	137.9 (22.1)	133.2 (21.1)	123.8 (21.2)	< 0.001
Mean diastolic blood pressure (SD), mmHg	78.8 (12.4)	78.7 (10.9)	75.5 (11.2)	< 0.001
Mean U-ALB Ratio (SD), mg/g	71.5 (298.1)	17.3 (43.7)	14.8 (32.6)	< 0.001
Mean U-Col4 Ratio (SD), μg/g	7.36 (6.2)	6.45 (3.7)	6.70 (4.2)	0.047

* All values are expressed as means (SD).

† Abbreviations are: eGFR, estimated glomelurar filtration rate; HbA1c, glycated hemoglobin; HDL, high-density lipoprotein; AST, aspartate amino transferase; ALT, alanine amino transferase; γ-GT, ganma-glutamyl transpeptidase; U-ALB Ratio, urinary albumin-creatinine ratio; U-Col4 Ratio, urinary type4 collagen creatinine ratio.

### Frequency of abnormal variables among the groups formed on the basis of the eGFR

The frequency with which abnormal values of BMI, total cholesterol, triglycerides, blood pressure, and ACR were measured differed significantly among the 3 groups. However, the serum levels of AST, ALT, and γ-GT did not differ among the groups (Table 2). Moreover, the rate at which abnormal values were recorded for HbA_1c _or U-Col4 excretion did not differ among groups.

**Table 2:** Frequency of abnormal variables among the groups divided by eGFR*

		eGFR, mL/min/1.73 m^2^		
		
variables	15--59	60--89	≥ 90	p value
Body mass index, %				
≥ 25 kg/m^2^	22.3	21.5	15.3	0.032
HbA_1c_, %				
>5.8%	13.1	9.1	9.3	0.336
Serum choresterol level, %				
>5.69 mmol/L (>220 mg/dL)	30	23.1	13.4	< 0.001
Serum triglyceride level, %				
>1.69 mmol/L (>150 mg/dL)	21.5	16.5	10.7	< 0.001
Serum HDL choresterol level, %				
<1.03 mmol/L (<40 mg/dL)	12.3	11.3	7.9	0.151
AST, %				
>40 U/L	3.8	4.9	4.1	0.737
ALT, %				
>40 U/L	3.8	7.9	7.1	0.235
γ- GT, %				
>60 U/L	12.3	14.4	16.7	0.404
Blood pressure, %				
Systoric ≥ 140 and diastoric ≥ 90 mmHg	48.5	37.3	25.4	< 0.001
U-ALB Ratio, %				
>17.9 mg/g	29.5	18.3	13.9	< 0.001
U-Col4 Ratio, %				
>8.2 (male), >9.4 (female) μg/g	20.2	16.6	18	0.541

* A χ^2 ^test was used for statistical comparisons.

† Abbreviations are: HbA1c, glycated hemoglobin; HDL, high-density lipoprotein;

AST, aspartate amino transferase; ALT, alanine amino transferase; γ-GT, ganma-glutamyl transpeptidase; U-ALB Ratio, urinary albumin-creatinine ratio; U-Col4

Ratio, urinary type4 collagen creatinine ratio.

### Prevalence of low eGFR and albuminuria with low eGFR and proteinuria

The prevalence of low eGFR for males and females was 6.6% and 9.4%, respectively. Among the individuals with low eGFR, the prevalence of microalbuminuria was 23.7% and 10.9% for males and females, respectively, and the prevalence of macroalbuminuria was 5.3% and 3.3% for males and females, respectively.

The prevalence of proteinuria with low eGFR was 5.3% and 4.9% and with eGFR >60 mL/min per 1.73 m^2 ^was 6.9% and 0.7% for males and females, respectively.

### Relationship between medical history and eGFR

A medical history of heart disease, hypertension, and hyperlipidemia was significantly associated with low eGFR (Table 3). However, a medical history of diabetes mellitus and renal disease was not associated with a low eGFR.

**Table 3:** Relationship between medical history and eGFR*

		eGFR, mL/min/1.73 m^2^		
		
		<60	≥ 60	
Medical History		(n = 130)	(n = 1424)	p value
Heart disease, %	Absent	87.7	93.8	
	Present	12.3	6.2	0.015
				
Cerebral infarction, %	Absent	95.4	96.8	
	Present	4.6	3.2	0.434
				
Hypertension, %	Absent	46.9	73.3	
	Present	53.1	26.7	< 0.001
				
Renal disease, %	Absent	95.4	97.9	
	Present	4.6	2.1	0.115
				
Diabetes mellitus, %	Absent	93.8	94.5	
	Present	6.2	5.5	0.689
				
Anemia, %	Absent	95.4	95.6	
	Present	4.6	4.4	0.825
				
Hepatic disease, %	Absent	95.4	98	
	Present	4.6	2	0.058
				
Hyperuricemia, %	Absent	96.2	98.5	
	Present	3.8	1.5	0.069
				
Hyperlipidemia, %	Absent	70	87.3	
	Present	30	12.7	< 0.001

*A χ^2 ^test was used for statisitical comparisons.

† Medical histories were previously diagnosed and self reported.

### Relationship between lifestyle and eGFR

Factors such as an alcohol intake, insufficient exercise, exercise habits, excess fat and salt intake, and weight changes from the age of 20 years were found to be significantly associated with a low eGFR. The percentages of drinkers and non-drinkers with low eGFR were 52% and 48% for males and 15% and 85% for females, respectively. However, smoking, eating at normal speed, and regular meal consumption were not significantly related to low eGFR (Table 4).

**Table 4:** Relationship between lifestyle and eGFR*

	eGFR, mL/min/1.73 m^2^			
	
Lifestyle		<60 (n = 130)	≥ 60 (n = 1424)	p value
Alcohol intake‡, %				
	(-)	59.2	49.8	
	(+)	40.8	50.2	0.044
Smoking habit§, %				
	(-)	70.8	68.4	
	(+)	29.2	31.6	0.622
Changing of Weight				
from 20 years old||, %	(a)	16.1	25	
	(b)	83.9	75	0.025
Insufficient exercise§, %				
	(-)	58.5	43.5	
	(+)	41.5	56.5	0.001
Exercise habit§, %				
	(+)	49.2	38.8	
	(-)	50.8	61.2	0.024
Eating speed**, %				
	(c)	72.3	65.4	
	(d)	27.7	34.6	0.122
Regular meal consumption§, %				
	(-)	6.9	12.6	
	(+)	93.1	87.4	0.067
Fat intake††, %				
	(-)	96.1	88.6	
	(+)	3.9	11.4	0.005
Salt intake‡‡, %				
	(e)	6.9	13	
	(f)	54.6	57.4	
	(g)	38.5	29.6	0.033

*A χ^2 ^test was used for statistical comparisons. † An eGFR ≥ 60 was used as the control group. ‡ (-):non-drinker, (+): drinker. §(+):having a lifestyle, (-): not having a lifestyle. || (a): not changed, (b): changed. ** (c): not faster, (d): faster.

†† (-): not excess, (+): excess. ‡‡ (e): light, (f): moderate, (g): heavy.

### Risk factors for different values of eGFR

To further investigate the factors influencing eGFR, a multiple regression analysis was applied to identify an optimal subset for the parameter that exhibits the strongest relationship with eGFR (Table 5). Factors such as age, U-Col4CR, medical history of hypertension and hyperlipidemia were found in the group with mild decreased eGFR (eGFR 60-89 mL/min per 1.73 m^2^) in a statistically significant manner (odds ratio: 1.07, 0.59, 1.47, and 2.12, respectively). However, in the group with low eGFR, age, gender, and a history of hypertension and hyperlipidemia were found to be associated factors (odds ratio: 1.21, 4.33, 2.49, and 7.86, respectively).

**Table 5:** Risk factors for different values of eGFR*

	eGFR, mL/min/1.73 m^2^			
	
	<60		60--89	
	
Variables	Odds ratio (95%CI)†	p value	Odds ratio (95%CI)†	p value
Ages (each 1 year older age)	1.21 (1.16--1.26)	0	1.07 (1.06--1.08)	0
Male	4.33 (1.37--13.74)	0.013	1.00 (0.65---.54)	0.983
BMI (kg/m^2^)				
(≥ 25 versus <25)	1.32 (0.59--2.97)	0.503	1.23 (0.85--1.79)	0.28
HbA_1c _(%)				
(>5.8 versus ≤ 5.8)	1.08 (0.37--3.19)	0.885	0.59 (0.33--1.06)	0.077
U-ALB Ratio (mg/g)				
(>17.9 versus ≤ 17.9)	1.03 (0.48--2.20)	0.94	0.80 (0.54--1.18)	
U-Col4 Ratio (μg/g)				0.255
(>8.2 versus ≤ 8.2 in males, >9.4 versus ≤ 9.4 in females)	0.51 (0.23--1.14)	0.101	0.59 (0.41--0.85)	0.005
Hypertension	2.49 (1.26--4.91)	0.009	1.47 (1.00--2.16)	0.049
Hyperlipidemia	7.86 (3.16--19.59)	0	2.12 (1.24--3.64)	0.006
Alcohol intake	1.11 (0.54--2.27)	0.779	0.87 (0.63--1.20)	0.403
Smoking habit	1.76 (0.57--5.39)	0.323	0.82 (0.55--1.24)	0.343

*An eGFR ≥ 90 was used as the control group. Multilogistic regression was used for statistical comparisons.

† Adjusted for age, gender, BMI, HbA_1c_, U-ALB Ratio, U-Col4 Ratio, Hypertension, Hyperlipidemia, Alcohol intake and Smoking habit.

‡ Abbreviations are: CI, confidence interval; BMI, body mass index; HbA1c, glycated hemoglobin; U-ALB Ratio, urinary albumin-creatinine ratio; U-Col4 Ratio, urinary type4 collagen creatinine ratio.

### Risk factors for low eGFR in the different age groups

We divided the participants into 2 groups on the basis of their ages. An eGFR ≥ 60 mL/min per 1.73 m^2 ^was considered as the control, the results of adjusted multivariate logistic regression analyses performed to identify the factors associated with low eGFR are shown in Table 6.

**Table 6:** Risk factors for low eGFR in different age groups*

	**<65 years**.		**≥ 65 years**.	
	
variables	Odds ratio (95%CI)†	p value	Odds ratio (95%CI)†	p value
Ages (each 1 year older age)	1.17 (1.02--1.34)	0.021	1.08 (1.04--1.12)	0
Male	1.94 (0.21--17.72)	0.557	2.97 (1.33--6.62)	0.008
BMI (kg/m^2^)				
(≥ 25 versus <25)	0.63 (0.13--3.02)	0.562	0.93 (0.56--1.54)	0.777
HbA_1c _(%)				
(>5.8 versus ≤ 5.8)	2.02 (0.16--26.24)	0.591	1.25 (0.58--2.68)	0.571
U-ALB Ratio (mg/g)				
(>17.9 versus ≤ 17.9)	6.28 (1.41--28.03)	0.016	1.14 (0.71--1.85)	0.582
U-Col4 Ratio (μg/g)				
(>8.2 versus ≤ 8.2 in males, >9.4 versus ≤ 9.4 in females)	0.50 (0.08--3.16)	0.463	0.98 (0.57--1.68)	0.943
Hypertension	2.13 (0.54--8.51)	0.283	1.62 (1.06--2.50)	0.028
Hyperlipidemia	9.43 (2.55--34.91)	0.001	1.97 (1.19--3.28)	0.009
Alcohol intake	0.59 (0.15--2.38)	0.455	1.66 (0.98--2.83)	0.059
Smoking habit	3.32 (0.45--24.38)	0.238	1.84 (0.87--3.91)	0.112

*Multilogistic regression was used for statistical comparisons.

† Adjusted for age, gender, BMI, HbA_1c_, U-ALB Ratio, U-Col4 Ratio, Hypertension, Hyperlipidemia, Alcohol intake and Smoking habit.

‡ Abbreviations are: CI, confidence interval; BMI, body mass index; HbA1c, glycated hemoglobin; U-ALB Ratio, urinary albumin-creatinine ratio; U-Col4 Ratio, urinary type4 collagen creatinine ratio.

In the group aged <65 years (younger group), factors such as higher age, high ACR, and hyperlipidemia were found to be associated with low eGFR in a statistically significant manner (odds ratio: 1.17, 6.28, and 9.43, respectively), whereas BMI, HbA_1c_, U-Col4CR, and hypertension were not associated with these diseases. However, in the elderly group (aged ≥ 65 years), male gender, hypertension, and hyperlipidemia were associated with low eGFR in a statistically significant manner (odds ratio: 2.97, 1.62, and 1.97, respectively), whereas BMI, HbA_1c_, ACR, and U-Col4CR were not. Thus, higher age and hyperlipidemia were the common risk factors, while the risk of having low eGFR was associated with ACR in the younger group and hypertension status in the elderly group.

## Discussion

In the present cross-sectional, community-based study, the prevalence of CKD (defined as an eGFR of <60 mL/min per 1.73 m^2^) was 8.4%. ACR is thought to be an independent associated factor for CKD, especially in individuals aged <65 years; however, U-Col4CR is not regarded as an associated factor for CKD despite its association with a decrease in the eGFR.

Measuring the GFR is regarded as the gold standard for assessing renal function. GFR is evaluated through the measurement of the urinary clearance of an ideal filtration marker such as inulin. Measurement of urinary clearance using exogenous markers is complex, expensive, and difficult to perform in routine clinical practice and cohort studies. We used an abbreviated version of the Modification of Diet in Renal Disease (MDRD) formula to estimate the GFR [13-15], and the CKD status was classified into 5 stages on the basis of the eGFR values [16]. In the present study, the prevalence of CKD (low eGFR) was 8.4%. Previous studies have reported the prevalence of CKD at stages 3-5 in several different populations. The prevalence was found to be 8.1% (white) and 3.8% (black) in 22,634 Americans aged 28-65 years (average age: 57.1 ± 11.6 years) in a study in which renal function had been estimated using the MDRD formula [9]. Further, the prevalence was found to be 6.8% in a study involving 3,499 Southeast Asian people aged 35-55 years using the MDRD formula [10]. In a community-based study on 7,316 Japanese people aged ≥ 30 years (average age: 52.4 years), the prevalence of this disease was estimated at 6.7% using the MDRD formula and at 8.5% using the Cockcroft-Gault equation [17]. The abovementioned results reveal that the variation in prevalence of CKD may depend on the participant characteristics, especially on their age and race, associated risk factors, and methods used for estimating the GFR. The Cr-based equations have been studied extensively and applied widely; however, the calibration of serum Cr assays to account for differences in the methods used has not been sufficiently standardized across laboratories.

The present study showed that lower eGFR values were likely to be related to high values of BMI, serum cholesterol, triglyceride, systolic and diastolic blood pressure, and ACR. The eGFR was inversely correlated to age (*p *< 0.001); this relationship between eGFR and age has been reported in a community study performed in the USA [18], an adult study conducted in the USA [19], and a cross-sectional health survey conducted in Norway [20].

Urinary albumin was used as a biomarker to detect early-stage diabetic nephropathy in type 1 [21] and type 2 diabetes [22]. Increased levels of urinary albumin excretion (UAE) may also indicate an unfavorable renal prognosis. However, screening for UAE is difficult because the cut-off range of ACR depends on the laboratory and sample collection methods used, such as collection at 24 h intervals, timed collection of samples, and spot collection. We calculated the normal cut-off range of ACR for 442 healthy adults (age, 20-70 years) who yielded normal results on both physical examination and laboratory analyses. In this study, the prevalence of abnormal ACR was significantly different among the 3 eGFR groups. The duration of the disease is an important factor to be considered for evaluating renal function. ACR measurement serves as a useful marker for the diagnosis of ESRD, especially during the early stage of proteinuria. ACR can be an independent associated factor for CKD patients, especially those aged <65 years. Our results indicate that ACR measurement is a good diagnostic marker for patients in the younger group with renal disease, but not for elderly patients with renal disease in the community-based study.

The present study showed that the participants with a low eGFR were older and more likely to be obese and hypertensive. Therefore, it is important to detect CKD and initiate treatment as early as possible in older populations in order to prevent the development of cardiovascular disease and ESRD [23]. The results of several cross-sectional studies have revealed that older age, hypertension, and diabetes mellitus are very strongly associated with a higher prevalence of elevated serum Cr level [24,25]. Another study reported that the mean HDL cholesterol levels were low, whereas both the systolic blood pressure levels and BMI were high in subjects with a low eGFR [26]. In this study, the presence of a medical history of heart disease, hypertension, and hyperlipidemia, was significantly related to low eGFR. These findings suggest that risk factors for cardiovascular disease may also be associated with decreased renal function. However, a history of diabetes mellitus and anemia was not related to low eGFR in this study. A probable explanation for this might be the self-reported medical history because the mean value of HbA_1_c and hemoglobin was not abnormal in this population.

Increasing lines of evidence suggest a relationship between lifestyle and the development and progression of CKD. In a previous case-control study, the effects of smoking (but not alcohol consumption) on CKD were identified [27]; however, our results did not find any appreciable risk among smokers. The association between smoking and CKD was verified by a dose-response trend, and an independent association was found to exist between excessive alcohol consumption (≥ 4 servings of alcohol per day) and CKD. When compared to the absence of smoking and alcohol consumption, smoking in combination with excessive alcohol consumption is reported to be associated with an almost fivefold increase in the odds ratio for developing CKD [28]. An excess of salt intake often causes hypertension and long-term consumption of fatty meals leads to hyperlipidemia. Excessive alcohol intake can influence nutritional deficiency. In the present study, a drinking habit and the consumption of fatty and salty meals, which are associated with metabolic disease, were found to be associated with low eGFR. These associations may be influenced by sex and age in participants. The amount of exercise and exercise habit (doing physical activity with sweat) might be recommended in the group with low eGFR. In contrast, smoking, eating speed, and regular meal consumption were not significantly related to low eGFR. These observations suggest that the type of lifestyle might influence the development and progression of CKD; however, there is no detailed information on the influence of lifestyle in this regard.

In diabetic nephropathy, mesangial matrix expansion and thickening of glomerular basement membrane are typical pathologic features, which are characterized by an increase of extracellular matrix (ECM) components. U-Col4 is one of the principal components of the renal ECM. It has been reported that U-Col4 excretion increases in patients with diabetic nephropathy [29], and the levels of U-Col4 may be a useful indicator to detect the early phase of diabetic nephropathy [12]. However, in the present study, we observed that U-Col4CR was in fact inversely associated with a mild decrease in eGFR (60-89 mL/min per 1.73 m^2^), and a borderline association was observed with diabetes (higher HbA_1c_). There was a higher prevalence of abnormal U-Col4 excretion and HbA_1c _with normal eGFR (≥ 90 mL/min per 1.73 m^2^) than with a mild decrease in eGFR. This suggests that subjects with an early phase of renal dysfunction and early diabetes with hyperfiltration might have a normal eGFR. There were not clear indications that U-Col4CR is a useful and early marker to screen or monitor kidney function in a community population. However, future investigations should focus on the causes of severe CKD. However, in low eGFR subjects, age, gender, and a history of hypertension and hyperlipidemia were found to be the associated risk factors.

Renal insufficiency increases in the elderly population; therefore, we classified the subjects into 2 groups on the basis of their ages. Both hyperlipidemia and ACR were found to be associated with a significantly increased risk for low eGFR (odds ratio, 9.43, and 6.28, respectively) in the younger group. This may be because many older participants may have received some treatment for their disease. Aggressive treatment administered to the younger group to ameliorate hyperlipidemia and improve the ACR during the early stages may prevent the decline of renal function. However, gender, hypertension, and hyperlipidemia were associated with a significantly increased risk for low eGFR (odds ratio, 2.97, 1.62, and 1.97, respectively) in the elderly group. These results indicate that gender (male) and hypertension can be important diagnostic factors for CKD in the elderly group.

Our study has several limitations. First, we did not directly measure the GFR; therefore, patients with at some stage of eGFR may have been wrongly diagnosed as having no kidney disease. Furthermore, we did not calibrate our serum creatinine measurements to the methods of the Cleveland Clinic, where the MDRD eGFR equation was derived. We validated the MDRD eGFR equation in a local population, and this also cause an overestimation in the prevalence of CKD. Second, the ACR and U-Col4CR values were obtained from a single survey because we used spot urine samples that were collected only once. Several measurements of both ACR and U-Col4CR should be performed to accurately determine the average concentrations. Third, prospective study designs should be considered for further research on the relationship between the amount of salt intake and eGFR because we did not measure salt intake accurately. Finally, the cross-sectional design of the present study makes it impossible to infer a causal relationship between CKD and its associated factors.

## Conclusion

Our community-based study revealed that the equation for predicting the GFR might be useful in clinical practice and for investigating kidney function. In addition, we propose that ACR could be a biomarker for estimating the risk of CKD in younger populations but not in older ones, while U-Col4CR is not an associated factor for either population. We also observed that associated factors for cardiovascular disease, such as heart disease, hypertension, and hyperlipidemia, were associated with renal dysfunction. Thus, we conclude that an early diagnosis of CKD and the identification of CKD-related associated risk factors by using the appropriate biomarkers may be useful for preserving renal function.

## Competing interests

The authors declare that they have no competing interests.

## Authors' contributions

NT, HA and TD were responsible for the design of this study, the interpretation of the results and writing this manuscript. TT provided advice in measuring urinary samples. KN, YI and YO were responsible for sample collection and questionnaires. JK and MK participated in performing statistical analysis and manuscript review. All authors read and approved the final manuscript.

## Pre-publication history

The pre-publication history for this paper can be accessed here:

http://www.biomedcentral.com/1471-2369/10/34/prepub
